# Estimating sources and sinks of malaria parasites in Madagascar

**DOI:** 10.1038/s41467-018-06290-2

**Published:** 2018-09-25

**Authors:** Felana Angella Ihantamalala, Vincent Herbreteau, Feno M. J. Rakotoarimanana, Jean Marius Rakotondramanga, Simon Cauchemez, Bienvenue Rahoilijaona, Gwenaëlle Pennober, Caroline O. Buckee, Christophe Rogier, C. J. E. Metcalf, Amy Wesolowski

**Affiliations:** 1 Institut Pasteur de Madagascar, 101 Antanarivo, Madagascar; 2 UMR 228 ESPACE-DEV (IRD, UM2, UR, UAG), Station SEAS-Ol, Saint-Pierre, Reunion, France; 3 Mathematical Modeling of Infectious Diseases Unit, Institut Pasteur, Paris, 75015 France; 4 Centre National de la Recherche Scientifique, URA3012, Paris, 75015 France; 5 Center of Bioinformatics, Biostatistics and Integrative Biology, Institut Pasteur, Paris, 75015 France; 6 Department of Epidemiology, Harvard School of Public Health, Boston, MA 02115 USA; 7 Center for Communicable Disease Dynamics, Harvard School of Public Health, Boston, MA 02115 USA; 8 Unité de recherche sur les maladies infectieuses et tropicales émergentes (URMITE), Paris, France; 9 Institute of Biomedical Research of the French Armed Forces (IRBA), Brétigny-Sur-Orge, France; 10 Department of Ecology and Evolutionary Biology, Princeton University, Princeton, NJ 08540 USA; 11 Woodrow Wilson School of Public Affairs, Princeton University, Princeton, NJ 08540 USA; 12 Department of Epidemiology, Johns Hopkins Bloomberg School of Public Health, Baltimore, MD 21231 USA

## Abstract

In areas where malaria epidemiology is spatially and temporally heterogeneous, human-mediated parasite importation can result in non-locally acquired clinical cases and outbreaks in low-transmission areas. Using mobility estimates derived from the mobile phone data and spatial malaria prevalence data, we identify travel routes relevant to malaria transmission in Madagascar. We find that the primary hubs of parasite importation are in a spatially connected area of the central highlands. Surprisingly, sources of these imported infections are not spatially clustered. We then related these source locations directly to clinical cases in the low-transmission area of the capital. We find that in the capital, a major sink, the primary sources of infection are along the more populated coastal areas, although these sources are seasonally variable. Our results have implications for targeting interventions at source locations to achieve local or national malaria control goals.

## Abstract (web summary)

Understanding the source of malaria outbreaks in low-transmission areas is important for controlling the disease. Here, the authors use mobile phone data to map malaria transmission in Madagascar, and are able to show that primary sources of infection in the capital city are found along populated coastal areas.

## Introduction

Identifying sources of malaria parasites, i.e., areas where humans may become infected and then, by travelling, subsequently introduce parasites into new populations, is a key component to the strategic deployment of interventions in environments with heterogeneous prevalence^1^. The rate of parasite importation is an important metric for assessing outbreak risk, and defines the feasibility of local elimination^2,3^. In Madagascar, human-mediated parasite importations have affected the landscape of malaria since at least 1902^4^ when infected rice cultivators imported parasites into the capital, an area with little local transmission but prone to outbreaks of the disease^5^. Outbreaks in the capital continue to be shaped by importation, with recent outbreaks attributed to residents traveling to endemic areas of the country. There is a growing concern that these importations may reestablish local transmission^6,7^. Understanding how travel drives the spatial epidemiology of malaria in Madagascar will be key to designing strategic malaria control efforts, disentangling local and non-local cases, and assessing the origin of imported clinical cases in the capital district to ultimately identify hotspots for targeted control.

Malaria transmission is spatially heterogeneous throughout the country^7^. The central highlands of Madagascar have low or no local transmission, whereas high-transmission areas along the coasts have prevalence estimates equivalent to mesoendemic areas of West Africa^7^. With such substantial heterogeneity in transmission, human travel and the subsequent movement of parasites plays an important role in defining malaria epidemiology, particularly in the low-transmission areas where travel by infected individuals undermines local control by reintroducing, and possibly sustaining, local disease transmission in zones without endemic transmission.

Identifying parasite movement networks has historically been difficult, particularly in settings with sparse data on human travel patterns^8–12^. While census data often provide the most reliable source of travel data^12^, Madagascar last conducted a national census in 1993 and the country currently lacks the infrastructure to collect national travel data relevant to disease transmission. Recently, mobility patterns quantified using mobile phone data has provided researchers with an alternative method to measure human travel^13,14^. Here, we combine travel patterns emerging from these data with spatial malaria infection prevalence data to identify routes of human-mediated parasite importations. We identify the relative importance of these routes and identify hotspots of malaria importations, for each delineating where parasites originated (sources) and where they are introduced (sinks). We assess the temporal stability of sources and sinks over the course of the year and identify areas that are temporally stable sources and that may yield the greatest impact from targeted malaria control.

To further ground our analysis, we related inferred parasite importation networks to clinical cases in the low-transmission capital city, Antananarivo. We identified likely source locations from the reported clinical cases and compared these projected sources to the source locations identified from the parasite importation network. We found that these two methods produce spatially similar results, with primary sources along the Southeast and Northwest coasts. Although limited in geographic scope, these results suggested that parasite importation networks may be directly related to clinical burden in certain low prevalence settings like the capital city, Antananarivo. We concluded by discussing what these patterns suggest for malaria control within Madagascar and across other countries with heterogeneous patterns of transmission.

## Results

### Mobility and malaria data in Madagascar

**Fig. 1:**
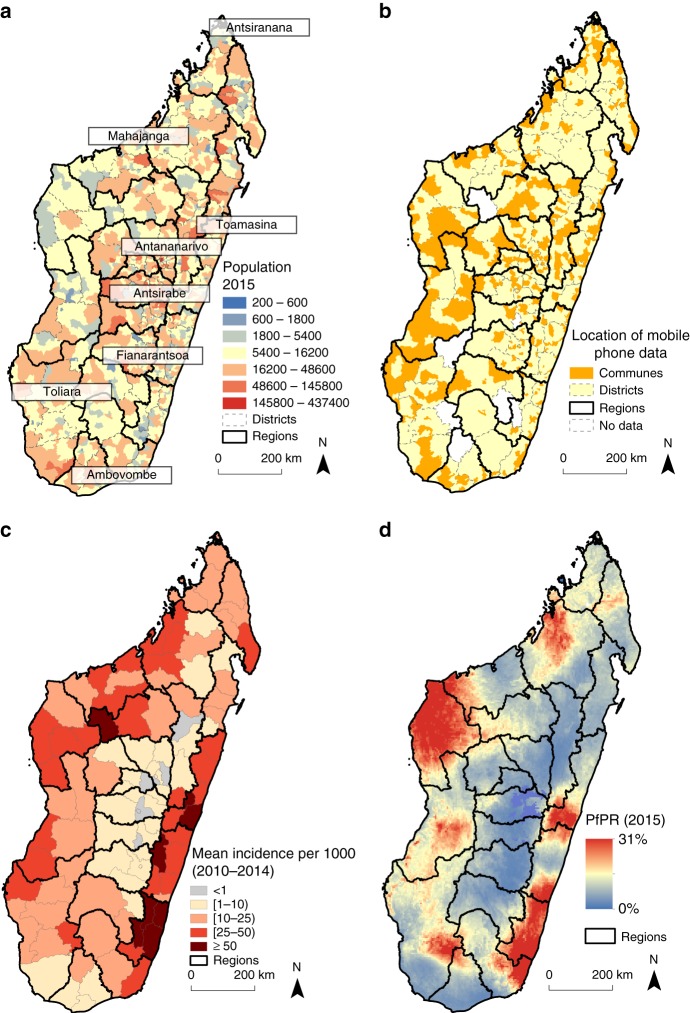
The population, mobile phone coverage, and malaria epidemiology of Madagascar. **a** Population estimates were obtained from National Institute of Statistic in Madagascar (INSTAT) and shown per commune with district (gray dashed lines) and region (black solid lines) boundaries shown. Major cities are also highlighted. **b** The majority of districts have at least one mobile phone tower (102/114 shown in yellow) with only 10% districts lacking coverage. Fewer communes had mobile phone coverage (367/1579 shown in orange). **c** The mean yearly reported incidence per 1000 is shown. Areas with the highest incidence (dark red) are primarily on the East coast, although both coasts had high incidence (red). Locations with very low incidence (grey) are primarily in the central highlands near Antananarivo. **d** The prevalence map (PfPR_2–10_) from the Malaria Atlas Project is shown. The prevalence map generally reflects the mean incidence with the highest PfPR_2–10_ values in the Southeast and North-Central coasts

We analyzed travel patterns between communes in Madagascar from 01 January 2015 to 30 June 2015 (see “Methods” section). Only 367 out of 1579 communes (23%, and 102 out of 114 districts–90%) had mobile phone coverage. Briefly, travel was defined as an overnight change in assigned mobile phone tower location (see “Methods” section). Although these communes cover the majority of districts, the geographic coverage limits our ability to comprehensively analyze the impact of mobility throughout the entire country. For example, coverage was limited on the southeastern area of the country, an area with high malaria transmission (see Fig. 1), which limits our ability to correctly assess the impact of this area on possible parasite movements in lower-transmission areas. However, in general, the communes with mobile phone coverage were more populated than those without coverage (see Fig. 1a, b, Supplementary Figures 1–2), although this likely only represents 31% of the population. In particular, communes within the capital city, an area of the country deemed at risk of malaria importations, did have mobile phone coverage and were included in the analysis.

These data were analyzed in conjunction with two sources of spatial malaria epidemiological data on *Plasmodium falciparum*. Reported, confirmed (via rapid diagnostic test or microscopy) cases of malaria provided by the Health Management Information System (HMIS) from 2010–2014 (Fig. 1c) and modeled prevalence estimates of the proportion of children aged 2–10 who were positive for *Plasmodium falciparum* parasites (PfPR_2–10_) in 2015 (Fig. 1d). Although the timeframes of the data do not overlap, both data sets are broadly consistent although the HMIS data shows substantial shifts in transmission over the time period and is likely underreported. Overall, transmission was highest along the East and Southwest coasts and lowest in the Highlands and Fringe areas (see Methods). Incidence was generally similar to prevalence, with the only exceptions in the more populated, low-transmission areas in the Highlands that reported a high number of cases (Pearson’s correlation coefficient average monthly incidence and mean district-level PfPR_2–10_ = 0.62, *p* < 0.001, Supplementary Figure 3). These data were used to inform an estimate imported parasites by individuals visiting a location and introducing these parasites (see Methods).

**Fig. 2:**
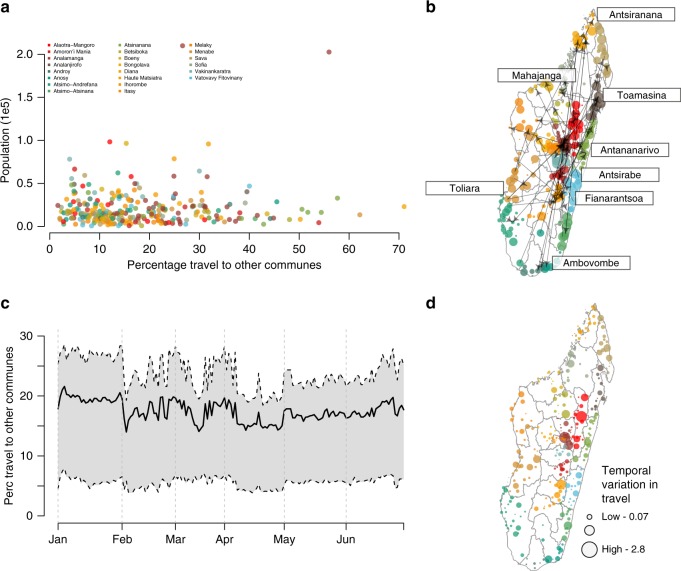
The mobility patterns in Madagascar. We quantified trips between communes (~59,000 routes between 367 communes) in Madagascar using mobile phone data (see “Methods” section). **a** The percentage of trips to other communes versus the population size is shown for communes colored by region. In the majority of districts, few trips were to other communes and the percentage was not biased spatially or by population. **b** The top quantile of trips between communes are shown as directed arrows, with colors for regions as in the previous figure. The majority of trips involved a major population center (labeled) including the central capital (Antananarivo). **c** Travel between communes was fairly consistent over the time series with dips in early February, mid-March, and April. **d** The coefficient of variation in the amount of travel originating from each commune showing that the largest areas are also most variable

Travel between Madagascar’s communes was highly local with 78% of travel within the same commune (min: 18.0, max: 99.6% range representing the variability amongst communes) and 86% within the same district (min: 24%, max: 99%) (see Fig. 2a, Supplementary Figure 4). For non-commune trips, the most traveled routes often included the capital, and more populated communes (Pearson’s correlation coefficient between amount of incoming travel and population: 0.749, Fig. 2b). Communes along the major road network were more likely to have travel between districts than within the same district (Supplementary Figure 5). Although the most common routes of travel remained stable over the course of the year, the total amount of travel varied temporally with small decreases during the end of the rainy season (early February, mid-March and April) when roads may be less accessible (Fig. 2c). We did not see a strong relationship between temporal variability and the origin commune’s population size (Fig. 2d, Pearson’s correlation coefficient: 0.017, *p* = 0.75, “Methods” section) with the largest variability displaying little spatial clustering (see Supplementary Figure 6). However, we did observe a small difference in trips from areas impacted (mean percentage of trips from communes impacted by the cyclone: 17%, 14–21%, median: 16%) by the cyclone in January 2015 vs. those that were not affected (mean percentage of trips from all remaining communes: 20%, 18–21%) (“Methods” section and Supplementary Figure 7).

### Estimating mobility and parasite importation networks

**Fig. 3:**
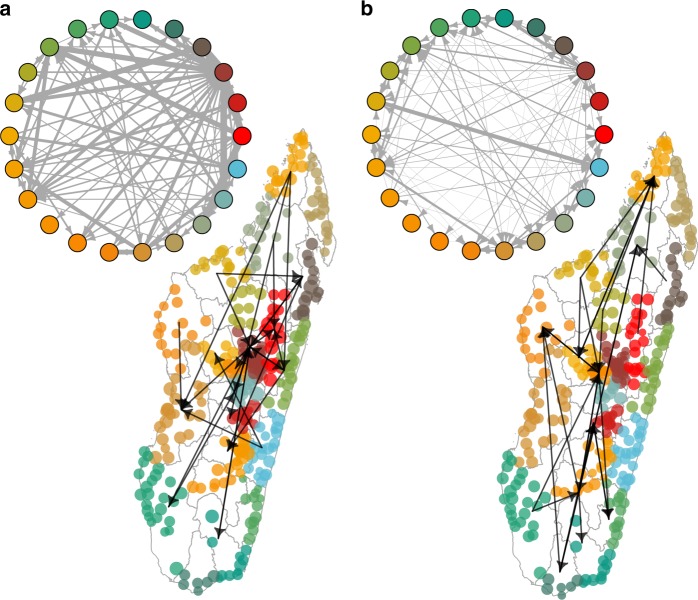
The travel and parasite importation networks. **a** The travel network between regions and top routes of travel between communes (*N* = 367 communes, colored by region as in Fig. 2). Parasite network importations with the top quantile of trips. **b** The top destinations for parasite importation included Atsinanana (East coast), Menabe (West coast), and Vatovay Fitovinany (East Coast), all regions located nearby the capital region (Analamanga)

Using these data, we built upon the mobility networks and estimated parasite importations throughout the country (Fig. 3a). Importations can occur in two ways: individuals who visit endemic areas may become infected during their trip, and potentially reintroduce parasites back to their resident commune. Conversely, individuals from high endemic areas may introduce parasites into low-transmission settings when they visit a new location. In both instances, imported infections may generate sporadic outbreaks, which have been observed throughout Madagascar, with travel noted as a possible cause^7,15^. However, the granularity of the mobile phone data did allow us to estimate round trips (see “Methods” section) and instead we have calculated a simplified parasite importation measure that describes an individuals’ probability of being infected while traveling to a new location to form a parasite importation network. The top routes of parasite importation were by individuals from the highlands region (Analamanga) (see Fig. 3b, Supplementary Data 1, clustering coefficient: 0.948) who traveled to the coastal regions of the north (Diana) and east (Atsinanana). Importantly, these routes of importation were not the same as the primary routes of travel (see Fig. 3a), where all regions were highly connected (generalized weighted clustering coefficient arithmetic mean: 1).

**Fig. 4:**
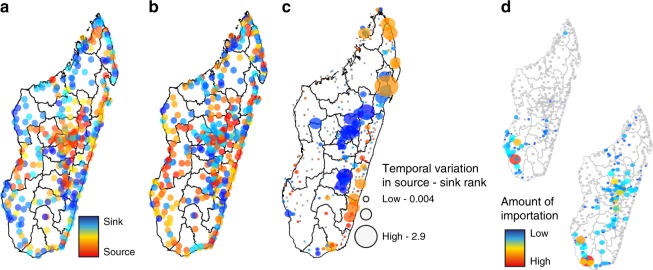
The sources and sinks of travel and parasite movement. We identified locations that were primarily sources versus sinks of **a** travel and **b** parasite movement for all communes included in the analysis. Sources and sinks of travel were geographically nearby, in contrast to the primary sink of parasites in the central highlands. **c** The temporal variability (coefficient of variation) in the source vs. sink rank over the time series of the data highlighting the most variable source and sink locations were along the East coast and in the central Highlands. **d** For two sample communes, Ampanihy and Betioky Sud, in the coastal region in Atsimo-Andrefana, the sink destinations are shown (locations with no importations are shown in grey). These two communes are similarly ranked sources. However, they differ both in terms of the number of sink locations and magnitude of importations at each sink location, suggesting that a more refined estimate of importation may be necessary to assess the impact of targeted interventions

Next, we identified primary sources and sinks of travel or malaria parasites throughout the country (see Methods). We found little spatial clustering of sources or sinks for travel alone, with many sources located near sinks, reflecting a large proportion of local travel (see Fig. 4a, Supplementary Figure 8). Key malaria parasite source locations were found along the coast where transmission is higher. In contrast to the sources of imported infections, we observed strong spatial clustering of sinks for malaria parasites (Fig. 4b, Supplementary Figure 8) in the Highlands, although these ranks are temporally variable (see Fig. 4c).

Clear differences between locations were observed with respect to the spatial distribution of import/export; some locations primarily exchanged parasites with a few focal areas, whereas others received or exported infections to a larger, more diffuse geographic area (Supplementary Figures 9–10). Even locations that exported the same number of infections, for example, may have exported the majority of infections to particular focal locations, or exported a more evenly distributed number of infections across a larger number of places. Similarly, sink locations varied with respect to the origin of imported infections; some received nearly all their imported parasites from a few locations, whereas others received parasites from across the country. Figure 4d shows an example of this, with two geographically nearby communes that have a similar source rank. However, one commune exports these parasites to a larger number and wider geographic distribution of sink locations where the number of infections exported to each one was quite low. In contrast, the commune’s exportations are to a small number of locations with a large amount of parasites spreading to each of these locations. Identifying these differences may be important for control programs since the effect of controlling transmission in source locations would vary spatially. Control in these two locations—one that may have a small impact on a large number of locations and another that would have a large impact on a smaller number of locations—would change the impact of targeted interventions.

### Parasite importations in the low-transmission capital city

**Fig. 5:**
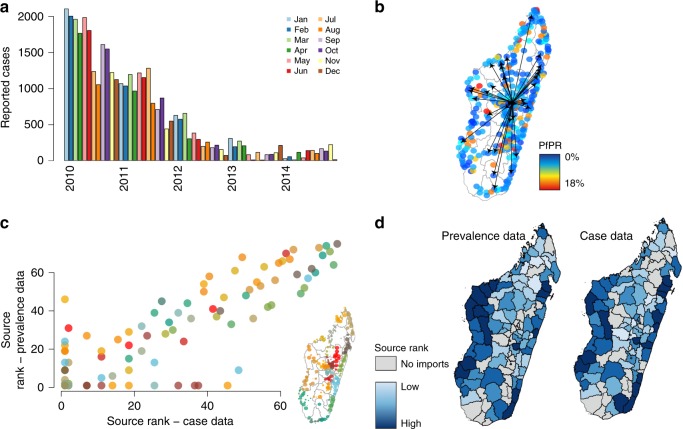
The source locations for cases in Antananarivo. **a** The reported cases from 2010 to 2014 per month to health facilities in Antananarivo. Overall, reported malaria has declined in the capital with the large number of cases reported in 2010–2013. **b** The routes of travel from Antananarivo to other communes with communes colored by their PfPR value (red = high, blue = low). **c** The source rank for all communes estimated from either the case data or the prevalence data (parasite network from Figs. 3, 4). **d** The comparison of top sources (dark blue) versus no importations (grey) to the cases in Antananarivo from the prevalence data versus the case data

Ultimately, these results would also directly relate to clinical cases, not simply the movement of parasites. However, the complexities of the life cycle of malaria, including the potential for long lasting infections and asymptomatic carriage, result in a non-linear relationship between prevalence and clinical burden. This means it is difficult to directly assess the impact of imported infections on clinical disease. However, in areas with low or no local transmission, we are able to estimate the likely source of these imported cases with previously developed spatial modeling methods that have been used in other low-transmission settings (see Methods)^16^. This approach relies only on clinical incidence data, which is likely underreported, and therefore provides a useful comparison to our parasite importation network, where estimates of the origin of reported clinical cases derived from i) the timing and amount of travel between the capital and other districts, ii) the generation time of malaria, and iii) the number of cases reported elsewhere in the country (see Fig. 5a, b and “Methods” section). We compare the results of these approaches for the capital district, Antananarivo, where local malaria transmission is primarily driven by imported infections^7,15^. Both methods identify a number of key sources (see Fig. 5c) along the East and West coasts. However, underreporting of the incidence data may also be spatially biased, implying that we are not properly identifying source locations. The overall agreement between both methods, including prevalence estimates that are likely less biased than incidence data, suggest that we are capturing general patterns of connectivity. The two methods primarily differ in the North-Central region, districts near Mahajanga, where key sources identified using the parasite importation network were not identified using the individual spatial modeling method, suggesting that although the overall pattern is similar, these two methods may differ in specific locations (Fig. 5d), although these results may be biased by irregular reporting of incidence data. These fine-scale differences may be more relevant when considering individual clinical cases in low-transmission settings and possible targeted intervention strategies related to these cases.

## Discussion

Sources and sinks of malaria parasites occur in settings when humans travel between locations with heterogeneous malaria prevalence^3,13,17^. An understanding of both the spatial epidemiology of malaria and mobility patterns of individuals is then necessary to identify routes of parasite importation. Here, we analyzed travel, malaria prevalence, and clinical cases in Madagascar to identify routes of travel and parasite importations throughout the country. We showed that the majority of trips are local, with few trips between districts in comparison to other countries analyzed using similar methods^13,14^. However, these results were limited by irregular mobile phone coverage, differential ownership rates, and only including data from a single provider. We did not extrapolate the data to non-coverage areas and only were able to analyze mobility patterns where the provider had coverage. However, we found little evidence of spatial clustering suggesting that although coverage is not homogeneous across the country, it is not spatially biased (Supplementary Figure 11). We identified a spatially homogenous parasite sink in the highlands of the country, with associated importations reflective of the importance of travel both to and between this region. Many source locations only contributed parasite importations to a small number of sink locations suggesting that the spatial targeting of these locations may have a localized, as opposed to country-wide effect. The main exception is areas on the major roadways and mid-sized port cities on both coasts, where targeted control may have more wide-spread secondary effects.

We then related the parasite importation network to inferred source locations based solely on the reported clinical cases in the low-transmission capital. We find that both methods identify similar sources, with the only notable exception the source location on the Northwest coast that was present in the parasite importation network analysis but not the specific analysis using the cases in Antananarivo. These results were based on the reported cases to health facilities that will be biased by accessibility to healthcare and the asymptomatic ratio/likelihood of clinical symptoms. However, we believe that the reported case data are able to provide a broad picture of the general incidence throughout the country. The case data was aggregated per month reducing our ability to accurately match cases based on the generation time. However, the agreement between both methods does suggest that source importations may be consistent into Antananarivo.

We did not take into account seasonality in transmission since the PfPR_2–10_ values are not traditionally calculated as seasonal measures. The interactions between seasonal variability in travel and the malaria season may affect these results if travel and transmission both seasonally increase above the baseline in particular locations. Moreover, these results are based on the mobility patterns of mobile phone owners, which may not be representative of the population. In particular, the age biases in mobile phone ownership likely imply that younger children are missing from these data. There is evidence that children are unlikely to travel without adults in Madagascar, which would imply that our results are biased upwards, but still capable of capturing the movement of children^18^. In addition, we assumed that reported clinical cases were reflective of ongoing transmission, although this may be less reliable in areas with poor access to health facilities and highly endemic areas that may have higher acquired immunity, reducing the probability an infected individual will experience clinical symptoms.

Recently, many parts of the country, including low-transmission areas, have experienced larger than expected outbreaks^7,15^. The driver of these outbreaks is unknown, however travel-mediated parasite introduction have been proposed as a possible cause^7,15^. Understanding how the relationship between parasite and human movements varies across the country and over the course of the year will be key to strategic malaria control. Further studies need to be performed to answer what part the importations of malaria parasite contribute in all reported cases especially in areas like Madagascar with highly heterogeneous malaria transmission and control.

## Methods

### Malaria in Madagascar

Madagascar’s population (23 million^19^ is divided into 22 regions that are further subdivided into 114 districts and 1,579 communes (Fig. 1a, Supplementary Figure 1). Malaria is a common cause of mortality and morbidity with an estimated ~475,000 cases per year^20^. The National Malaria Control Program (NMCP) divides Madagascar into five malaria strata: Highlands, Fringe, South, East, and West^7^. These strata are defined by their location and malaria epidemiology: Highlands/Fringe—low-transmission, sporadic outbreaks; South—unstable transmission, unstable seasonality; East—high and perennial transmission; and West—high and seasonal transmission. We utilized two sources of malaria data: reported cases and spatial parasite rate estimates. We analyzed reported cases of uncomplicated malaria provided by the Health Management Information System (HMIS). Monthly, RDT confirmed cases per reporting district were analyzed from 2010 to 2014 (see Supplementary Data 2). We also used 1 km by 1 km gridded parasite rate, PfPR_2–10_, estimates from the Malaria Atlas Project^21^. Mean PfPR_2–10_ values per district ranged from 0.56 to 18% (mean: 5.9%) with the lowest values in the central highlands and highest values along the East and West coasts. Additional analyses using the maximum and minimum PfPR_2–10_ values per district were also used to capture variability in values within a district.

### Mobility patterns

We quantified mobility estimates using mobile phone data across Madagascar provided by Orange Madagascar (see Fig. 1b). From 01 January 2015 to 30 June 2015, each subscriber was assigned a commune based on the location of the first mobile phone tower per day. At the time of data collection there were three mobile phone providers within the country. Missing data from other mobile phone operators were not adjusted for in the analysis, and hence we can only estimate importations within the area where the operator has coverage.

Trips between communes were counted when an individual’s commune location was different on subsequent days. If these two locations were the same, then the subscriber was identified as staying in the same commune. Trips and stays were provided to the authors as percentages and then transformed to an estimated number of trips based on the commune population and mobile phone ownership. We used the reported mobile phone ownership rates in the most recent Malaria Indicator Survey (MIS) (Supplementary Data 3)^22^. In order to protect the anonymity of subscribers, individual travel trajectories were unavailable. As a result, we were only able to assume that a subscriber’s resident commune was the previous day’s location. We were also unable to estimate each trip’s duration and assumed an exponential distribution for the duration of trips. We assumed that a district was likely impacted by the cyclone if it was within the path of Tropical Storm Chedza, which began on the West coast to Antananarivo (Supplementary Figure 7)^23^.

### Quantifying malaria parasite importations

We quantified malaria importations based on the flow of individuals between locations. A simple transmission model was built upon the travel network between communes. Parasite importation was estimated to describe the process where individuals in a location (*i*) may acquire parasites while traveling elsewhere (*j*). Each subscriber was assigned a commune based on the previous day’s first used tower location. The amount of importation by individuals from location *i* to location *j* was calculated per trip:

$${\rm{Import}}(i,j) = 1 - \left( {1 + \alpha \ast \beta \ast {\mathrm{dEIR}}_j \ast l} \right)^{ - \frac{1}{\alpha }}$$

As in previous work we assumed fixed parameter values for *α* and *β* with *α* = 4.2, an estimate of heterogeneous biting rates of mosquitos during a trip, *β* = 1/20 a probability of infection given an infectious bite based on^24,25^. Mean district-level PfPR_2–10_ values were converted to the entomological inoculation rate (EIR), the number of mosquito bites per night times the proportion of those bites positive for sporozites (Supplementary Data 1). We used a previously defined relationship between EIR and PfPR_2–10_: EIR = −1.573 + 7.74* PfPR_2–10_ with EIR = 0 when PfPR_2–10_ = 0. Further analyses were conducted using the minimum and maximum PfPR_2–10_ for each district to capture the variability in transmission within these units (see Supplementary Data 4, 5). We converted these to a mean daily measure, dEIR, assuming a log-normal distribution^26,27^ and *l* is the duration of each trip. Since we did not have access to individual movement trajectories and only aggregated trip counts, trip durations were drawn from an exponential distribution with the rate set to 1/*λ*, with *λ* equal to 5 and present values from the mean estimate. In the remaining analysis mean values are presented. The import values were summed over the total number of trips between all pairs of communes (or districts, regions) for consecutive days.

We measured the clustering of the parasite importation networks using the generalization for weighted networks of the clustering coefficient.

### Identified sources and sinks of parasites

We ranked each commune based on the amount of parasite importation. Sources are locations that emit a large number of parasites—and thus are ranked highly in terms of exportation. Conversely, sinks are locations that import a large number of parasites—and are thus ranked highly in terms of importation. We assessed the temporal variability in a location’s source-sink rank over the course of the year by calculating the coefficient of variation (CV) of their rank. Communes with the highest CV are those that change their rank the most over the course of the year, and do not necessarily correspond to those with the highest rank. We assessed the spatial clustering of source and sink locations using ncf R package (Supplementary Figures 6, 8). We analyzed the amount of clustering within the parasite networks using the method by Opsahl et al.^28^.

### Inferring the source location of cases in Antananarivo

The parasite importation networks provide an understanding of the flow of parasites throughout the entire country. However, this approach is limited in two important ways: (1) it does not directly relate possible parasite importations with clinical cases and (2) it cannot disentangle the burden associated with non-locally acquired cases and locally acquired cases since the importation dynamics are inherently intertwined with the local dynamics.

We determined if cases reported in the capital district, Antananarivo Renivohitra commune (referred to as: Antananarivo), were acquired from the following (1) a local transmission chain or (2) from transmission outside of Antananarivo using previously developed methods to identify the relationship between individual cases in a low-transmission setting^16^. We note, however, that the case reporting system in low-transmission settings may be unreliable and remains subject to inherent biases in clinical case reporting associated with malaria^29^.

For each reported case in Antananarivo, we estimated the likely source location based on the clinical cases reported elsewhere in the country, the connectivity, and the generation time of malaria. The probability that for a clinical case in Antananarivo, *c* on day *t* was infected by a case in location *j*, *T* days previously given the amount of travel *h* (*j*) from Antanarivo to *j* is:

$$P\left( {c,t} \right) = h\left( j \right) \ast G\left( {T - t} \right) \ast {\rm{Inc}}(j,T)$$

where *G*(*T − t*) is the generation time (see below) and Inc(*j*,*T*) is the incidence of malaria in location *j* at time *T*. We assumed that the reported number of cases was reflective of ongoing transmission in these areas, and that may be untrue if there is a high proportion of asymptomatic individuals, non-local cases in other districts, and differential environmental suitability for transmission. Since the mobile phone data and case data do not completely temporally overlap, we used the average number of trips between districts. The generation time of malaria was estimated using Churcher et al.^2^ that includes the incubation periods of both the mosquito and second host and assumes the individual is treated. Borrowing the notation from and estimated values from Churcher et al.^2:^

$$G\left( x \right) = X_1pX_2nX_3Y_1Y_2Y_3$$

where *X*_1_ ∼ *lN*(2.380,0.254) is the human prepatient period, *X*_2_ ∼ Gamma(1.19,0.16) is the human duration of infectiousness, *X*_3_ ∼ *M*(9.5) is the mosquito duration of infectiousness, *Y*_1_ ∼ Gamma(3.42,1.08) is the human patency to symptoms period, *Y*_2_ ∼ *M*(3.05) is the human symptom to treatment period, *Y*_3_ ∼ *M*(3) is the treatment to parasite clearance period, *p* = 9*d* is the human latency period, and *n* = 10*d* is the mosquito latency period. Since the reported case data was on available on a monthly interval, we aggregated the generation time per month that likely misses many of the temporal heterogeneities in transmission.

### Code availability

Relevant code is provided in the supplementary information.

## Electronic supplementary material

Supplementary Information

Description of Additional Supplementary Files

Supplementary Data 1

Supplementary Data 2

Supplementary Data 3

Supplementary Data 4

Supplementary Data 5

## Data Availability

Reported cases of malaria, estimates of mobile phone ownership, and model output data files are available as supplementary files. Due to privacy agreements, access to aggregated versions of the mobile phone data are available upon request to the authors (awesolowski@jhu.edu).
