# Supplementary material for: Estimating sources and sinks of malaria parasites in Madagascar
Source: Nat Commun. 2018 Sep 25;9:3897. doi: 10.1038/s41467-018-06290-2 (PMC6156502; doi:10.1038/s41467-018-06290-2)
Supplement: Supplementary file 1 — Supplementary Information [file 41467_2018_6290_MOESM1_ESM.docx]

**Supplementary Information**

| 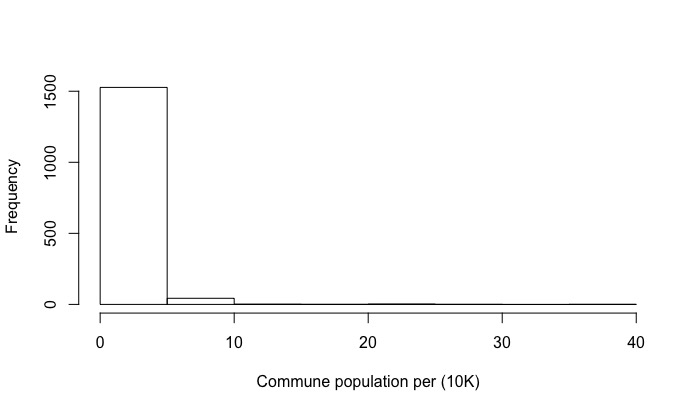 |
| --- |
| **Supplementary Figure 1: The distribution of commune populations.** The population per commune was estimated using data from Worldpop ([www.worldpop.org)](http://www.worldpop.org)). The average population was ~15,000 persons (90% quantile interval: 2,398 – 42,323), although this varied greatly where the smallest commune only includes 203 individuals and the largest with a population above 364,000. |

|  |
| --- |
| **Supplementary Figure 2: The population of districts with and without mobile phone coverage.** In the mobile phone data analyzed, we compared the populations of communes without mobile phone coverage (by the provider) versus those with mobile phone coverage. The majority of communes in Madagascar did not have mobile phone coverage (width of the boxplot), but were less populated than those with coverage (error bars represent the lower and upper quartiles). In total, 1,222 communes did not have mobile phone coverage with an average commune population of: 13,512 (90% quantile interval: 2,186 – 36,187). Of the remaining communes (N = 356) which did have coverage, the average population is: 21,281 (90% quantile interval: 3,297 – 51,271). |

|  |
| --- |
| **Supplementary Figure 3: The average monthly incidence from 2010-2015 versus PfPR per district (N=114).** |

|  |
| --- |
| **Supplementary Figure 4: The distribution of average non-commune travel.** For each commune, we calculated the average daily percentage of trips to other communes (labeled non-commune). |

| 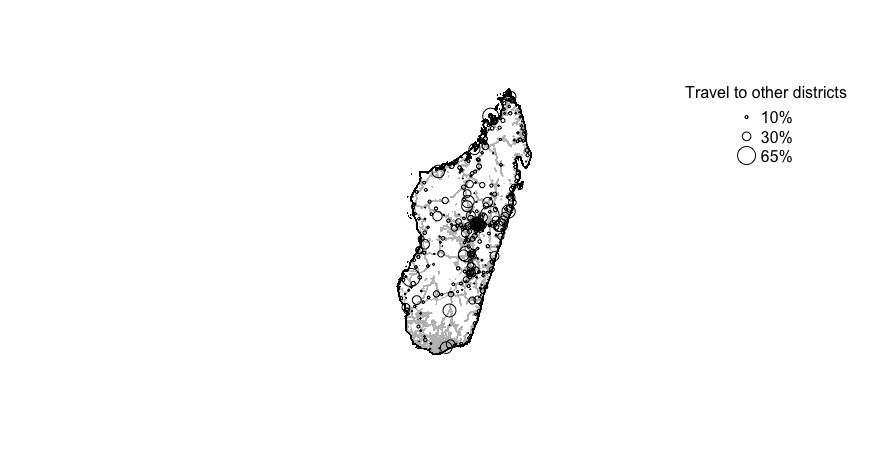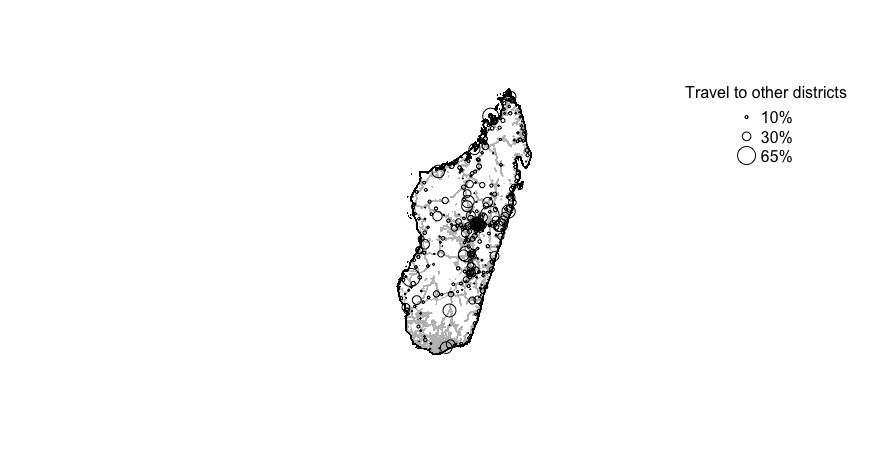 |
| --- |
| **Supplementary Figure 5: The travel to other districts.** For each commune (N=367), we calculated the average percentage of trips to other districts versus the same district. Areas nearby major roads (shown in grey) have a higher percentage of trips to other districts. |

|  |
| --- |
| **Supplementary Figure 6: The spatial correlation between temporal variability in travel per commune**. For each commune, we calculated how much travel varied over the course of the year (the coefficient of variation of the percentage of trips to other communes). We identified spatial clustering using a spline correlog (R package: ncf) and the longitude and latitude of each commune’s centroid (with ranges provided from 100 bootstrapped samples). We did not see a strong spatial signature or clustering of high temporal variability locations. |

| **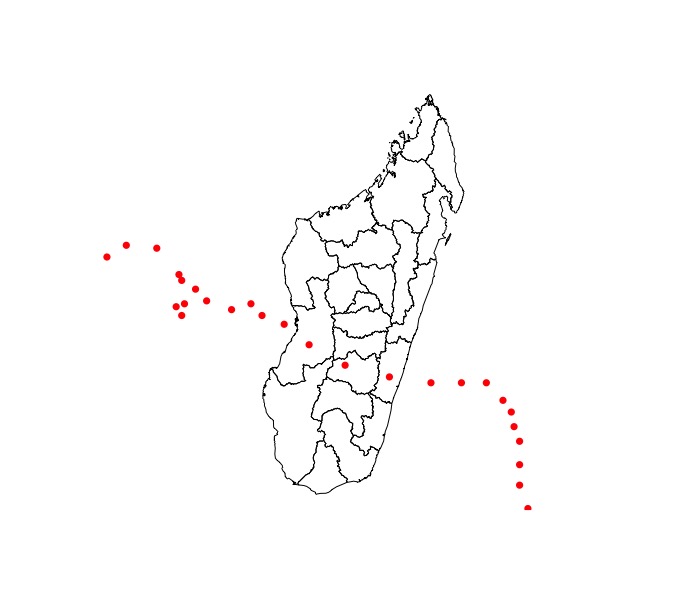** |
| --- |
| **Supplementary Figure 7: The path of tropical storm Chezda. (**[**https://journals.ametsoc.org/doi/abs/10.1175/2009BAMS2755.1**](https://journals.ametsoc.org/doi/abs/10.1175/2009BAMS2755.1)**)** |

|  |
| --- |
| **Supplementary Figure 8: The spatial clustering of sources and sinks.** For each commune (N=367), we calculated the amount of spatial clustering of source or sink ranks using a spine correlog (R package: ncf) and the longitude and latitude of each commune’s centroid (with ranges provided from 100 bootstrapped samples). |

|  |
| --- |
|  |
|  |
| **Supplementary Figure 9: The number of destinations per commune.** For all pairs of communes (~56,000 routes), we calculated the average number of daily trips. We then calculated the number of destinations from each commune where there was on average at least 1, 10, or 20 trips per day. |

|  |
| --- |
| **Supplementary Figure 10: The communes with a large number of destinations.** For each commune, the number of destinations where on average at least 10 trips were taken per day was calculated. Communes with at least 70 destinations are shown with an x, all communes are colored according to their region and sized by the amount of travel as in (Figure 2). |

|  |
| --- |
| **Supplementary Figure 11: The spatial clustering of communes with mobile phone tower coverage.**  Using the centroid of communes with mobile phone tower coverage included in this study and a Ripley’s K-function, we find little evidence of spatial clustering. |

**Data availability**

*Mobile phone coverage:* Mobile phone coverage data per province were obtained from the Madagascar Malaria Indicator Survey (2016) <https://dhsprogram.com/what-we-do/survey/survey-display-505.cfm>.

*Malaria Plasmodium falciparum prevalence estimates: Plasmodium falciparum* prevalence estimates were obtained from the Malaria Atlas Project <https://map.ox.ac.uk/explorer/#/explorer>.

*Reported clinical cases of malaria:* These data are provided as an additional data file.

**Additional data files**

Data file 1: The estimated amount of parasite importation between communes using the district-level mean P*f*PR value (origin: row, destination: column).

Data file 2: Reported cases by the National Malaria Control Programme.

Data file 3: Mobile phone coverage estimates from the Malaria Indicator Survey.

Data file 4: The estimated amount of parasite importation between communes using the district-level maximum P*f*PR value (origin: row, destination: column).

Data file 5: The estimated amount of parasite importation between communes using the district-level minimum P*f*PR value (origin: row, destination: column).

**Additional Source Code to run in R**

### Code for: Estimating sources and sinks of malaria parasites in Madagascar

### Code to calculate importations between locations. This code assumes that you have a matrix of trip counts between sublocations and the PfPR values for each sublocation. The mvt.matrix and pfpr.matrix must have the same number of rows that are matched.

est.import<-function(mvt.matrix, pfpr.values, lambda = 5, alpha = 4.2, beta = 1/20){

nn.loc<-nrow(mvt.matrix)

eir.per.loc<-sapply(pfpr.values, function(x) (-1.573) + (7.74*x*100))/365

import.matrix<-matrix(,nn.loc,nn.loc)

for(ii in 1:nn.loc){

eir.res<-eir.per.loc[ii]

for(jj in 1:nn.loc){

eir.dest<-eir.per.loc[jj]

numb.trips<-ceiling(mvt.matrix[ii,jj])

trip.durations<-rexp(numb.trips, 1/lambda)

est.import<-1-(1+alpha*beta*eir*trip.durations)^(-1/alpha)

import.matrix[ii,jj] = sum(est.import,na.rm=T)

}

}

colnames(import.matrix)<-rownames(import.matrix)<-rownames(mvt.matrix)

}

### Code to calculate the importations into the capital using the reported clinical cases

### start: generation interval

rtrunc <- function(n, spec, a = -Inf, b = Inf, ...)

{

x <- u <- runif(n, min = 0, max = 1)

x <- qtrunc(u, spec, a = a, b = b,...)

return(x)

}

qtrunc <- function(p, spec, a = -Inf, b = Inf, ...)

{

tt <- p

G <- get(paste("p", spec, sep = ""), mode = "function")

Gin <- get(paste("q", spec, sep = ""), mode = "function")

tt <- Gin(G(a, ...) + p*(G(b, ...) - G(a, ...)), ...)

return(tt)

}

simulate.generation.time<-function(treat){

## units in days

p = 9; n = 10

numb.samples = 5000000

X1 = rlnorm(numb.samples, meanlog = 2.38, sdlog = 0.254)

X2 = rgamma(numb.samples, 1.19, 0.016)

X3 = rtrunc(n = numb.samples, 'exp', rate = 1/9.5, a = 10, b = 30)

Y1 = rgamma(numb.samples, 3.42, 1.08)

Y2 = rexp(numb.samples, 1/3.05)

Y3 = rexp(numb.samples, 1/3)

if(treat == TRUE){

valid = ifelse((X2 + p) <= (Y1 + Y2 + Y3), 1, 0)

results<-X1+X2+X3+p+n

return(results[which(valid == 1)])

}

else{

results<-X1+X2+X3+p+n

return(results)

}

}

sim.treat.gen<-simulate.generation.time(TRUE)-19

sim.untreat.gen<-simulate.generation.time(FALSE)-19

make.daily.prob.dist<-function(values){

hist.values<-hist(values, breaks = seq(0,2*365))

prob.values<-hist.values$counts/sum(hist.values$counts)

plot(prob.values)

return(prob.values)}

make.monthly.prob.dist<-function(values){

hist.values<-hist(values, breaks = seq(0,3*365))

prob.values<-hist.values$counts/sum(hist.values$counts)

month.breaks<-c(seq(1,3*365,by=30), 365*3)

bb1<-c(0,sapply(1:(length(month.breaks)-1), function(x) sum(prob.values[month.breaks[x]:month.breaks[(x+1)]])))

return(bb1)}

daily.prob.treat.gen<-make.daily.prob.dist(sim.treat.gen)

daily.prob.untreat.gen<-make.daily.prob.dist(sim.untreat.gen)

monthly.prob.treat.gen<-make.monthly.prob.dist(sim.treat.gen)

monthly.prob.untreat.gen<-make.monthly.prob.dist(sim.untreat.gen)

### end: generation interval

single.loc.spatial.wt<-function(case.matrix, pfpr.values, mvt.matrix, focal.loc, generation.time){

nn.loc<-dim(case.matrix)[1]

time.steps<-dim(case.matrix)[2]

gg<-rev(generation.time) ## make most recent most likely

mvt.test<-mvt.matrix[,focal.loc]/sum(mvt.matrix[,focal.loc], na.rm=T)

mvt.matrix[focal.loc] = 0

origin.cases.no.focal<-case.matrix

origin.cases.no.focal[is.na(origin.cases.no.focal)]<-0

origin.cases.no.focal[focal.loc,] = 0

final.origin<-matrix(0,nn.loc,(time.steps-2))

for(jj in 3:time.steps){

focal.numb.cases<-ifelse(is.na(case.matrix[focal.loc,jj]), 0, case.matrix[focal.loc,jj])

origin.cases<-case.matrix[,((jj-2):jj)]

origin.w.gen<-t(apply(origin.cases.no.focal, 1, function(x) gg*x))

origin.w.mvt<-origin.w.gen*mvt.test

origin.w.mvt<-rowSums(origin.w.mvt)

origin.w.mvt<-origin.w.mvt/sum(origin.w.mvt, na.rm=T)

likely.origin<-sample(1:nn.loc, focal.numb.cases, prob = origin.w.mvt, replace = TRUE)

numb.occ.case<-sapply(1:nn.loc, function(x) length(which(likely.origin == x)))

final.origin[,(jj-2)] = numb.occ.case

final.origin.pfpr[,(jj-2)] = numb.occ.pfpr

}

return(c(list(final.origin = final.origin, final.origin.pfpr = final.origin.pfpr)))}
