# Supplementary material for: Estimating sources and sinks of malaria parasites in Madagascar
Source: Nat Commun. 2018 Sep 25;9:3897. doi: 10.1038/s41467-018-06290-2 (PMC6156502; doi:10.1038/s41467-018-06290-2)
Supplement: Supplementary file 2 — Description of Additional Supplementary Files [file 41467_2018_6290_MOESM2_ESM.pdf]

Description of Additional Supplementary Files

File Name: Supplementary Data 1
Description: The estimated amount of parasite importation between communes using the districtlevel mean PfPR value (origin: row, destination: column).

File Name: Supplementary Data 2
Description: The estimated amount of parasite importation between communes using the districtlevel maximum PfPR value (origin: row, destination: column).

File Name: Supplementary Data 3
Description: The estimated amount of parasite importation between communes using the districtlevel minimum PfPR value (origin: row, destination: column).

File Name: Supplementary Data 4
Description: Mobile phone coverage estimates from the Malaria Indicator Survey.

File Name: Supplementary Data 5
Description: Reported cases by the National Malaria Control Programme.
